# Research on autonomous walking performance and electromechanical characteristics of mining double-track chassis

**DOI:** 10.1371/journal.pone.0312096

**Published:** 2024-12-03

**Authors:** Zeren Chen, Yongpeng Wang, Fei Yang, Ruibin Li, Peng Han, Duomei Xue

**Affiliations:** 1 College of Mechanical Engineering, Taiyuan University of Technology, Taiyuan, Shanxi, China; 2 Taiyuan Heavy Machinery Group Co., Ltd, Taiyuan, Shanxi, China; 3 State Key Laboratory of Engine and Powertrain System, Weichai Power Co., LTD, Weifang, Shandong, China; 4 College of Chemical Engineering and Technology, Taiyuan University of Science and Technology, Taiyuan, Shanxi, China; Buckinghamshire New University - High Wycombe Campus: Buckinghamshire New University, UNITED KINGDOM OF GREAT BRITAIN AND NORTHERN IRELAND

## Abstract

To study the autonomous walking performance and corresponding electromechanical characteristics of unmanned mining equipment under different slopes, turning radii, and ground conditions. Firstly, the autonomous walking systems based on PID, fuzzy PID, and BP PID, in this paper, are constructed, and then the electromechanical coupling simulation is carried out to analyse autonomous walking performance and electromechanical characteristics of mining double-track chassis under different working conditions. Finally, the feasibility of the autonomous walking system based on fuzzy PID is verified by the path-tracking experiment. The results show that the autonomous walking performance of the autonomous walking system based on the fuzzy PID is the best. Under the soft ground, the current, voltage, and load torque are all increased to varying degrees due to the sinking phenomenon of the crawler, but the driving speed is reduced, and when mining double-track chassis makes large-radius turns, the autonomous walking system based on the BP PID can also be given priority with a path deviation within 0.1 m.

## Introduction

With the continuous development of automation technology and the constant increase in demand for mineral resources [1, 2], unmanned mining equipment (UME) has become a new development direction in the mining field. Autonomous walking performance and electromechanical characteristics as the key performance of UME will directly affect its operational reliability. The electromechanical characteristics described here mean that the operating process of the UME is essentially a process of interactive coupling between the mechanical and electrical systems. In this process, the voltage and current of the electrical system, the load torque and the driving speed of the mechanical system are the key electromechanical characteristics.

He *et al*. [3] presented a two-layer controller for accurate and robust lateral path tracking control of highly automated vehicles, which is based on a linear time-varying model predictive control (MPC) and a radial basis function neural network proportion-integral-derivative (PID). The results showed that the proposed controller could achieve a good level of path tracking accuracy. Tian *et al*. [4] proposed a hierarchical adaptive control framework based on MPC and PID. The results showed that the proposed hierarchical adaptive control framework can enhance roll stability for intelligent heavy vehicles. The path-following problem of an unmanned surface vehicle with unknown dynamics and unmeasured velocities is addressed by Qu *et al*. [5]. They proposed a heading-surge guidance scheme based on the traditional light-of-sight guidance. For a six-degrees-of-freedom underactuated underwater vehicle, Yu *et al*. [6] proposed an improved three-dimensional line-of-sight guidance law in the kinematic layer.

Precup *et al*. [7] proposed two applications of Grey Wolf Optimizer algorithms to a path planning problem and a Proportional-Integral-fuzzy controller tuning problem. To address a path following the problem of Wave Glider along a predefined path in the horizontal plane, Wang *et al*. [8] developed a fuzzy adaptive PID controller. Besides, for the difficulty of modelling and control of four-wheel electric vehicles, Dogan *et al*. [9] introduced a robust and adaptive position control based on Proportional-Integral control. Shi *et al*. [10] proposed a path tracking controller that can handle discontinuous trajectories and sudden orientation changes for hTetro. When the model contains important uncertainties, Salt *et al*. [11] used model-based dual-rate (MBDR) control for UGV path tracking. The results showed that MBDR outperformed Dual-rate inferential control. Rayguru *et al*. [12] developed a robust-observer based sliding mode controller to fulfill the motion control task in the presence of incomplete state measurements and sensor inaccuracies for cleaning robots, and the controller is successfully validated through numerical simulations. Finally, Rao *et al*. [13] used image processing technology for path tracking of smart cars. The results showed that the control methods were feasible and improved the real-time and stability of the control.

It can be seen that the current research mainly focuses on underwater robots [14], cleaning robots [12], and four-wheel electric vehicles [9], etc. However, there are few studies on path tracking methods of UME. The typical characteristics of UME are large volume and large inertia, and the walking chassis is mostly crawler, so its movement flexibility is low and autonomous walking is difficult. Secondly, because PID is capable of stabilizing complex systems and has gained wide acceptance due to their advantages of simple structure, ease of design, and low cost in implementation [15, 16]. The real-time and robustness of fuzzy control are very good. BP neural network is a multi-layer feedforward network trained by error back propagation. Gradient search technology is used to minimize the error mean square error between the actual output value and the expected output value of the network [17]. Therefore, the corresponding autonomous walking systems are designed based on these three algorithms. Finally, there is little attention to the electromechanical characteristics of autonomous walking processes, which have an important influence on path tracking dynamic performance, resulting in large fluctuations in the initial tracking of mining equipment [18, 19].

Therefore, the UME autonomous walking systems based on PID, Fuzzy PID, and BP PID are first introduced in this article, and then the electromechanical coupling simulation is carried out to compare the autonomous walking performance and electromechanical characteristics of mining double-track chassis under different working conditions. On this basis, the tracking effect of the autonomous walking system based on fuzzy PID is verified through actual experiment.

## Materials and method

### Double-track chassis

**Fig 1:**
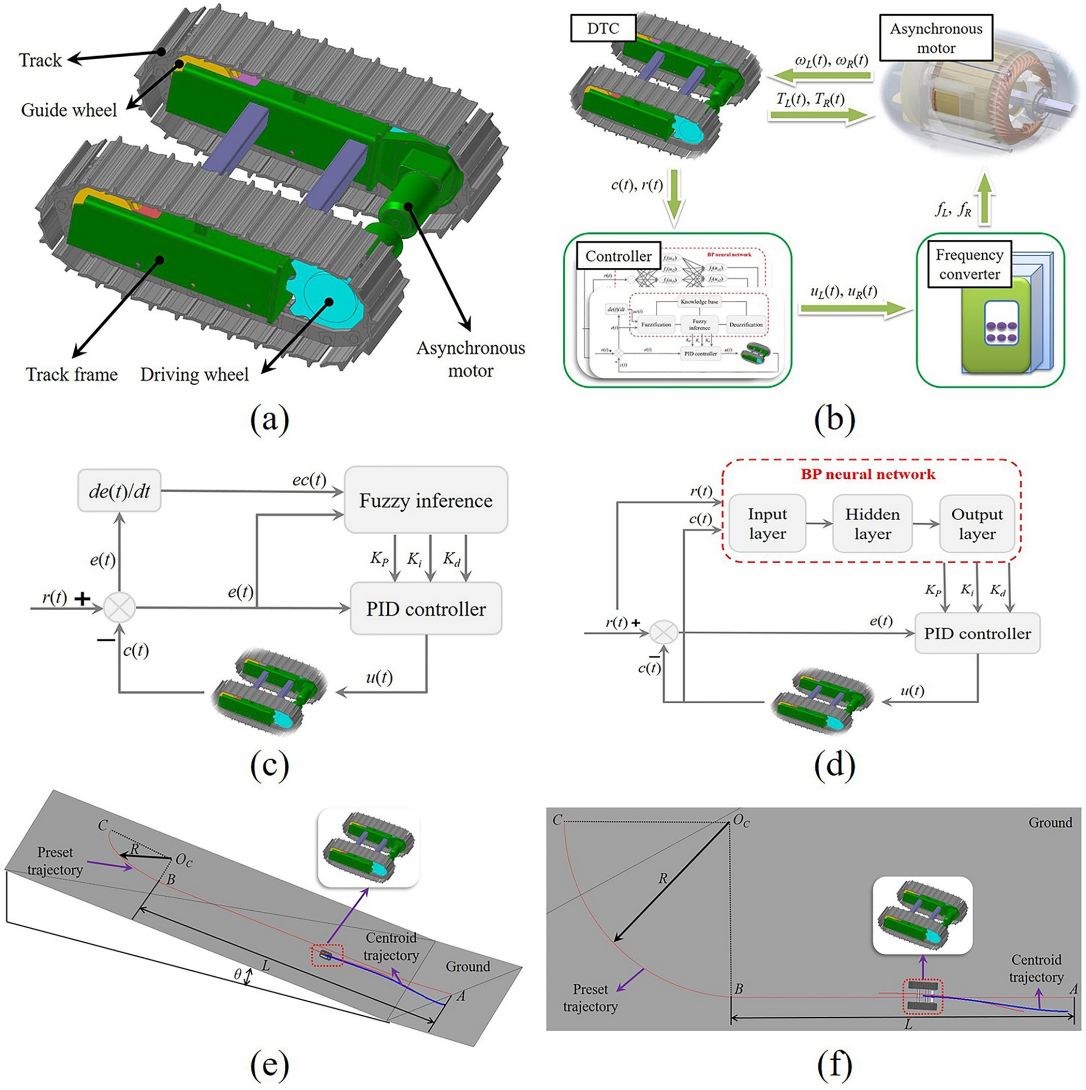
(a) Schematic diagram of mining double-track chassis. (b) Autonomous walking system. (c) Schematic diagram of fuzzy PID controller. (d) Schematic diagram of BP PID controller. (e) Slope preset trajectory. (f) Turn preset trajectory.

At present, mining equipment such as electric shovels, excavators, and bulldozers are mainly driven by mining double-track chassis [20–22]. During the autonomous walking process, the operating state of the UME is mainly controlled by adjusting the speed of the crawler drive motors on both sides. Therefore, this paper takes the mining double-track chassis (DTC) as the UME dynamic model. As shown in Fig 1(A), the DTC mainly includes tracks, guide wheels, driving wheels, and asynchronous motors. During the traveling of the DTC, the asynchronous motor transmits power to the driving wheels through the reducer, and the driving wheels drive the corresponding crawlers to produce winding motion, thereby realizing the operation of the DTC. The corresponding structural parameters and physical parameters are listed in S1 Table of S1 File.

### Autonomous walking system

The autonomous walking system described in this article mainly includes DTC, asynchronous motor, frequency converter, and controller, as shown in Fig 1(B). The function of DTC is to transmit the desired position *r*(*t*) and actual position *c*(*t*) information to the controller, at the same time, transmit the load torques (*T*_*L*_(*t*), *T*_*R*_(*t*)) to the asynchronous motor. The controller obtains the path deviation according to the input information and then transmits the control information (*u*_*L*_(*t*), *u*_*R*_(*t*)) to the frequency converter according to the control law. The function of the frequency converter is to convert the control signal into the power supply frequency (*f*_*L*_, *f*_*R*_) of the asynchronous motor and transmit them to the asynchronous motor based on the V/f control principle. The function of the asynchronous motor is to calculate the expected speeds (*ω*_*L*_(*t*), *ω*_*R*_(*t*)) based on the obtained load torque and power supply frequency and transmit the speeds to DTC.

#### Fuzzy PID controller

The PID control algorithm calculates the control amount of the path deviation *e*(*t*) through proportional, integral, and differential modules. Its corresponding control functions are mainly as follows: (1) The proportional module adjusts the input and output of the controlled system in proportion to make the trajectory deviation show a steady trend; (2) Integral module is mainly used to eliminate the steady-state error; (3) Due to the inertia of the controlled system and the lag of the control system signal acquisition, the controller needs to have a certain “forward-looking”, the differential module can predict the trend of the path deviation and react in advance. The corresponding control law can be expressed as Eq (1), according to literatures [16, 23, 24], the values of proportional coefficient *K*_*P*_, integral coefficient *K*_*i*_, and differential coefficient *K*_*d*_ are 100, 0.05, and 200 respectively.


$$u(t)=K_{p}e(t)+K_{i}\int_{0}^{t}e(t)dt+K_{d}\dot{e(t)}$$ (1)

Fuzzy PID control is a control method produced by the fusion between PID control and fuzzy control. Its control principle is shown in Fig 1(C). The path deviation *e*(*t*) and its change *ec*(*t*) are used as the input signal of the fuzzy PID controller, the proportional coefficient *K*_*P*_, integral coefficient *K*_*i*_ and differential coefficient *K*_*d*_ is adjusted by fuzzy inference. The defuzzification method is the centroid. Compared with PID control, it is more robust and suitable for complex nonlinear time-varying systems. The ranges of *e*(*t*) and *ec*(*t*) are determined based on actual condition of DTC, whose ranges are respectively [–350, 350] mm and [–30, 30] mm/s. The corresponding membership function is shown in S1(a) and S1(b) Fig of S2 File.

The ranges of *K*_*P*_, *K*_*i*_, and *K*_*d*_ are determined based on the comparative tuning method, whose ranges are respectively [90, 130], [0, 0.4], and [100, 180]. The corresponding membership function is shown in S1(c)-S1(e) Fig of S2 File. The fuzzy rule for the fuzzy PID controller is designed based on the previous description and simulation tests, listed in S2 Table of S1 File.

#### BP PID controller

BP PID controller includes a BP neural network and the PID algorithm. As shown in Fig 1(D), BP neural network is used to adjust *K*_*P*_, *K*_*i*_, and *K*_*d*_ in real-time and transmit them to the PID algorithm. In this paper, the BP neural network is a typical neural network structure that includes an input layer, a hidden layer, and an output layer [25]. Here, the ranges of *K*_*P*_, *K*_*i*_, and *K*_*d*_ are determined based on simulation tests, whose ranges are respectively [0, 120], [0, 0.05], and [0, 100]. The activation functions of the hidden layer and the output layer are *f*_1_(*u*_*i*_(*t*)) and *f*_2_(*u*_*o*_(*t*)), respectively, and the corresponding formulas are as follows:

$$\left\{ \begin{array}{l} f_{1}(u_{i}(t))=\frac{e^{u_{i}(t)}-e^{-u_{i}(t)}}{e^{u_{i}(t)}+e^{-u_{i}(t)}}\\ f_{2}(u_{o}(t))=\frac{e^{u_{o}(t)}}{e^{u_{o}(t)}+e^{-u_{o}(t)}} \end{array}\right.$$ (2)

Where, *u*_*i*_(*t*)—inputs of the hidden layer, *u*_*o*_(*t*)—inputs of the output layer.

### Ground simulation setting

This paper focuses on the study of the path tracking performance of the path tracking system and the electromechanical characteristics of the DTC under different slopes and different turning radii. Meanwhile, the ground type is set to the hard road surface and dry sand soft road surface. The hard ground can’t cause crawler sinkage, but the crawler sinkage occurs on the soft one. The common point of the two is that they can reflect the slippage and slippage of the crawler. For hard ground, the contact force between the track and the ground can be expressed as Eq (3).

$$\left\{ \begin{array}{l} F_{n}=k\delta^{m_{1}}+c\vec{e}{\left|\dot{\delta }\right|}^{m_{2}}\delta^{m_{3}}\\ F_{f}=-\mathrm{sign}(v)F_{n}\mu_{d} \end{array}\right.$$ (3)

Where, *F*_*n*_—contact normal force, *F*_*f*_—friction force, *k*—stiffness, *c*—damping coefficient, *δ* - penetration, $\dot{\delta }$ - time differentiation of the penetration, *μ*_*d*_ - dynamic friction coefficient, $\vec{e}$ - unit direction vector of $\dot{\delta }$. sign(*v*) - symbolic function of running speed (*v*), *m*^1^, *m*2—non-linear contact force exponents, *m*^3^—indentation damping effect exponent.

For soft ground, according to Bekker’s theory [26], the contact force between track and ground can be expressed as Eq (4). The corresponding ground parameters are listed in S1 Table of S1 File.

$$\left\{ \begin{array}{l} p=(k_{c}/b+k_{\phi })z^{n}\\ \tau =(C+p\tan\phi )(1-e^{-j/K}) \end{array}\right.$$ (4)

Where, *p*—pressure, *τ*—shear stress, *b*—width of contact area, *k*_*c*_—cohesive deformation modulus, *k*_*φ*_—friction deformation modulus, *z*—sinkage, *n*—soil deformation index, *C*—cohesion, *φ—*angle of internal shearing resistance of the terrain, *j*—shear displacement, *K*—shear deformation modulus.

### Simulation under different slopes

In view of the fact that in the actual work process of UME, the working environment is uneven and there is a climbing phenomenon, but the slope should not be too large. Because the large slope can easily cause the UME to roll over. For the large UME, even a small slope can cause a big change in its path tracking performance. At the same time, the corresponding electromechanical characteristics are also changed. Therefore, this section sets the slope *θ* to 0°, 5°, and 10°, as shown in Fig 1(E). The red line ABC is the preset trajectory, AB is a straight line segment, the length *L* is 32 m, BC is a 1/4 arc, and the radius *R* is 10 m. The starting point is point A, and the endpoint is point C. The initial position deviation is 2.13 m. The blue line is the trajectory of the DTC centroid.

### Simulation under different turning radii

In the actual working process of the UME, it is inevitable to make a turn. However, due to the large volume and weight of the UME, slip and slip phenomena occur between the DTC and the ground, resulting in a deviation between the actual turning radius and the theoretical turning radius. Therefore, this section focuses on the path tracking performance and corresponding electromechanical characteristics of the UME path tracking system when turning on flat ground. Here, the theoretical turning radius *R* is set to 8 m, 10 m, and 12 m, respectively. Fig 1(F) shows the steering trajectory diagram. The preset trajectory is the curve ABC, where the AB section is a straight line section, the length *L* is 15 m, BC is a 1/4 arc, point A is the starting point, and point C is the endpoint. The blue line is the centroid trajectory of the DTC. The initial position deviation is 0.5 m.

### Experimental verification

Based on the previous analysis results, the tracking effect of the autonomous walking system based on fuzzy PID is verified through actual tests in this section. The experiment device is a double-track test platform, as shown in S2 Fig of S2 File, which consists of a Real-Time Kinematic (RTK) positioning system, frequency inverter, NI cRIO controller, battery, computer, and a double-track chassis. The RTK positioning system is used to obtain real-time position information of the dual-track chassis and is powered by the battery. The function of the computer is to carry the autonomous walking control program and transmit the control signal to the cRIO controller. cRIO controller used for the lower computer is to control the frequency inverter, and it is used to control the crawler motor. Taking into account the limitation of the actual size of the test site and the operating capacity of the double-track test platform, this article has made corresponding adjustments to the test path, but the shape is similar to the one in Simulation under different turning radii Section, where the length of the straight line is 13 meters, the turning radius is 10 meters, the slope is zero degrees, the running speed is set to 0.1 m/s, and the other parameters are consistent with those in Simulation under different turning radii Section.

## Results and discussion

### Analysis under different slopes

#### Hard ground conditions

**Fig 2:**
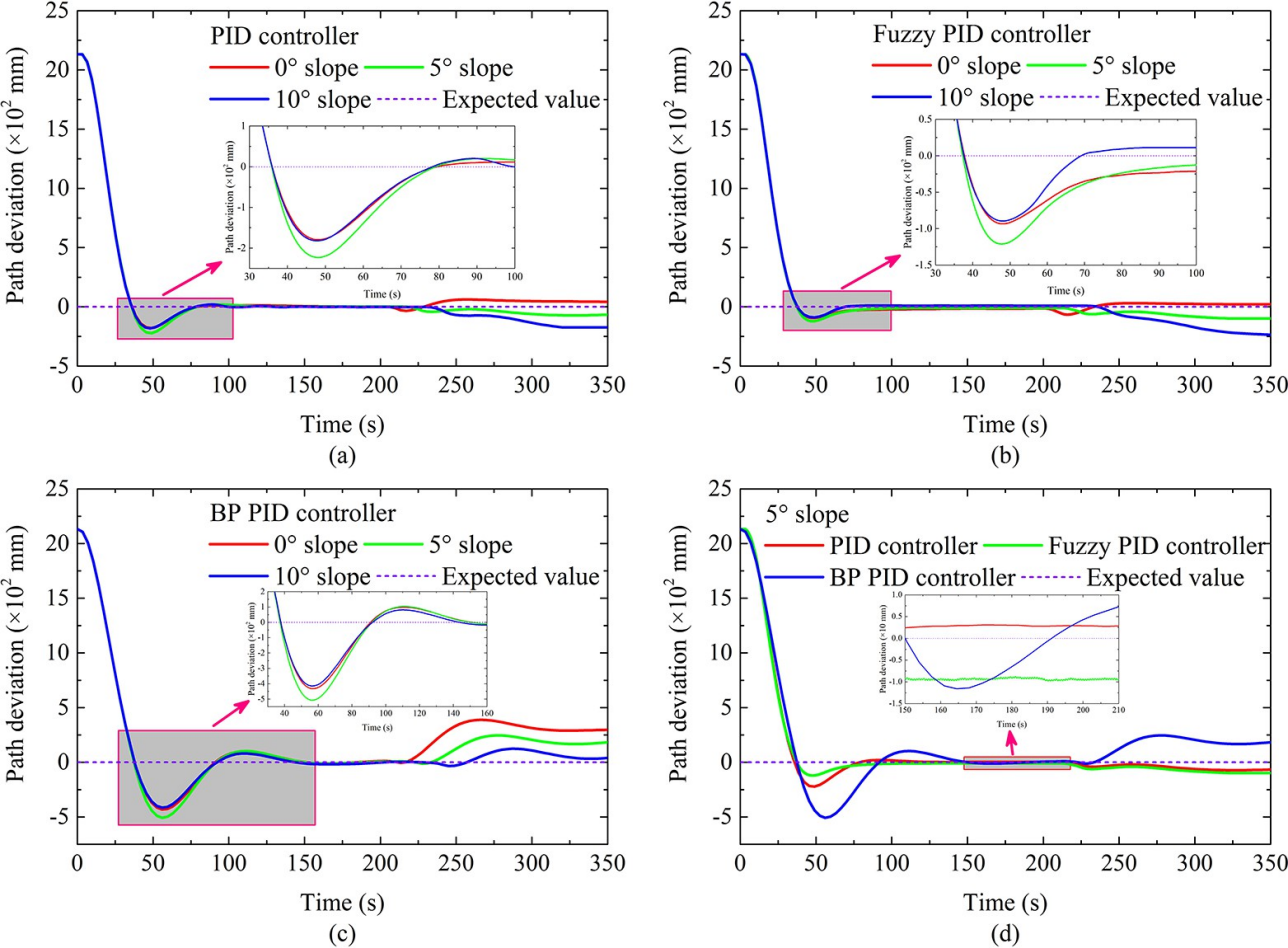
Autonomous walking performance under climbing of hard ground.

The autonomous walking performance under hard ground conditions is shown in Fig 2. When the running time is from 0 s to 200 s, the DTC is in the AB straight-line autonomous walking stage. When the running time is greater than 200 s, the DTC enters the BC curve autonomous walking stage. As far as the overshoot of the AB section is concerned, it can be seen that the overshoot based on fuzzy PID is least affected by the slope. On the contrary, the overshoot based on BP PID is most affected by the slope, this shows that the fuzzy PID controller has better adaptability. As far as the straight-line running time is concerned, as the slope increases, the running time becomes longer. This is mainly because the increase in slope causes the slippage between the crawler and the ground to become serious, which in turn leads to a decrease in the actual running speed of the DTC. As for the path deviation of the BC section, as the slope increases, the path deviation changes from a positive value to a negative value, which shows that the DTC moves from the outside to the inside of the curve, it is also caused by the aggravated slip between the crawler and the ground. Furthermore, it can be seen that the curve path deviation based on BP PID changes significantly, which indicates that the BP PID controller has a weaker perception of path deviation, which may be caused by the underfitting and the slow convergence speed of the BP neural network. In terms of response time, the response time based on fuzzy PID and PID is relatively small, and the response time based on BP PID is relatively long. Finally, the linear overshoot based on fuzzy PID is the smallest, and all linear steady-state errors are similar, which shows that the autonomous walking performance of the autonomous walking system based on fuzzy PID is the best.

**Fig 3:**
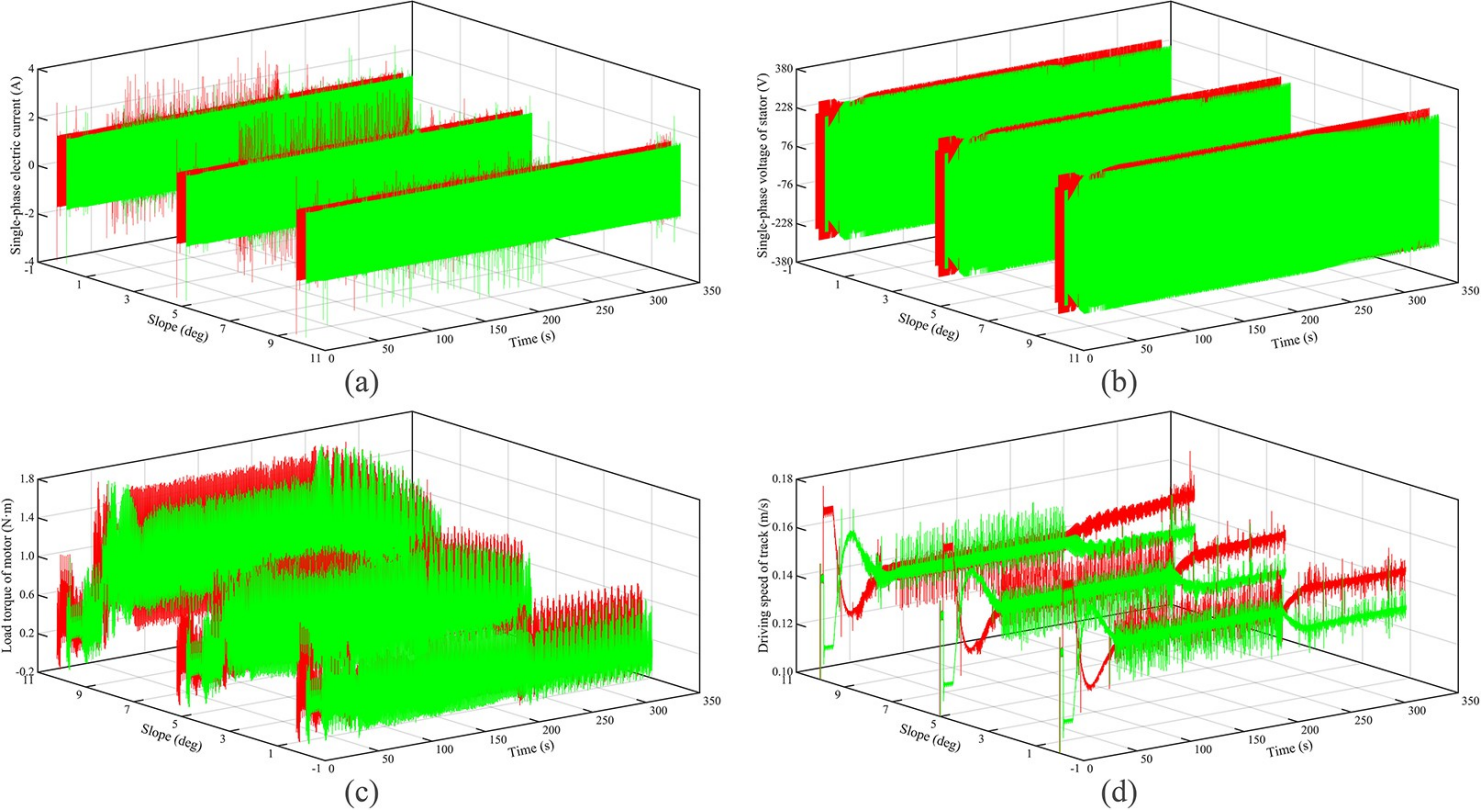
Electromechanical characteristics under climbing of hard ground.

The electromechanical characteristics of the autonomous walking system based on the fuzzy PID are shown in Fig 3. Red curves represent the left track, and green curves represent the right track. It can be seen that as the slope increases, the motor current, voltage, and load torque all increase to varying degrees, but the theoretical driving speed of the crawler is reduced. The main reason is that with the increase of the slope, the load of the DTC along with the road surface increases, resulting in the motor load torque to increase, so the theoretical driving speed of the crawler is reduced. This causes the mechanical power of the motor to decrease and the thermal power to increase, which is why the current and voltage is increased. Secondly, when the running time is from 0 s to 50 s, the DTC turns right and then left. It can be seen that the current, voltage, and load torque of the left crawler motor first increase and then decrease, while the right crawler changes the opposite. This shows that when the DTC turns, the load and power of the outer motor will become larger. When the running time is the 50 s to 200 s, the DTC basically maintains a straight-line driving state. At this time, the electromechanical characteristics of the left and right crawlers are approximately the same. This is because the load and the running state of both crawlers of the DTC are the same. When the running time is greater than 200 s, DTC enters the curve tracking stage. As the slope increases, the current difference between the left and right crawler motors does not change significantly, the voltage difference increases, while the load torque gap decreases, which shows that the steering of the DTC is mainly realized by the variation of voltage. At this time, relative to turning, the slope becomes the main influence of the motor load torque.

#### Soft ground conditions

**Fig 4:**
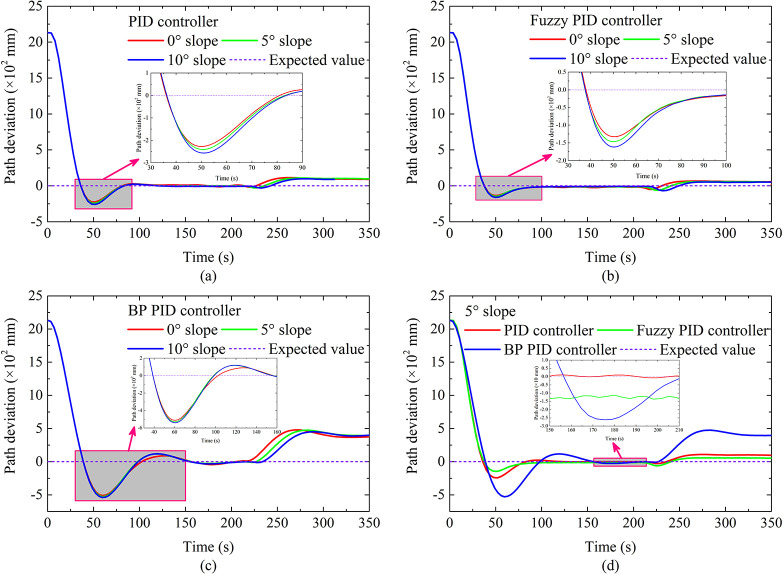
Autonomous walking performance under climbing of soft ground.

The autonomous walking performance under soft ground conditions is shown in Fig 4. When the running time is from 0 s to 210 s, the DTC is in the AB straight-line autonomous walking stage. When the running time is greater than 210 s, the DTC enters the BC curve autonomous walking stage. As far as the overshoot of the AB section is concerned, it can be seen that the overshoot based on fuzzy PID is least affected by the slope. On the contrary, the overshoot based on BP PID is most affected by the slope, which is consistent with the results on hard ground. The difference is that the overshoot is increased by about 50 mm. The reason is that there is a lowering of the steering flexibility of the DTC due to the pressure-sinkage effect of the crawler under the soft ground condition, which makes DTC unable to adjust the driving status in time.

As far as the straight-line running time is concerned, as the slope increases, the change in straight-line running time is consistent with the results on hard roads, and the corresponding values become larger compared to hard roads. This is mainly because the increase in slope causes the slippage between the crawler and the ground to become serious, coupled with the sinkage effect of the track, which in turn leads to a decrease in the actual running speed of the DTC. As for the path deviation of the BC section, as the slope increases, the path deviation is basically unchanged, but the corresponding values become larger compared to hard ground, which is caused by a lowering of the steering flexibility of the DTC. In terms of response time, the response time based on fuzzy PID and PID is relatively small, and the response time based on BP PID is relatively long, which may be caused by the slow convergence speed of the BP neural network. Furthermore, the linear overshoot based on fuzzy PID is the smallest, this is consistent with the results on hard ground, and all linear steady-state errors are similar.

**Fig 5:**
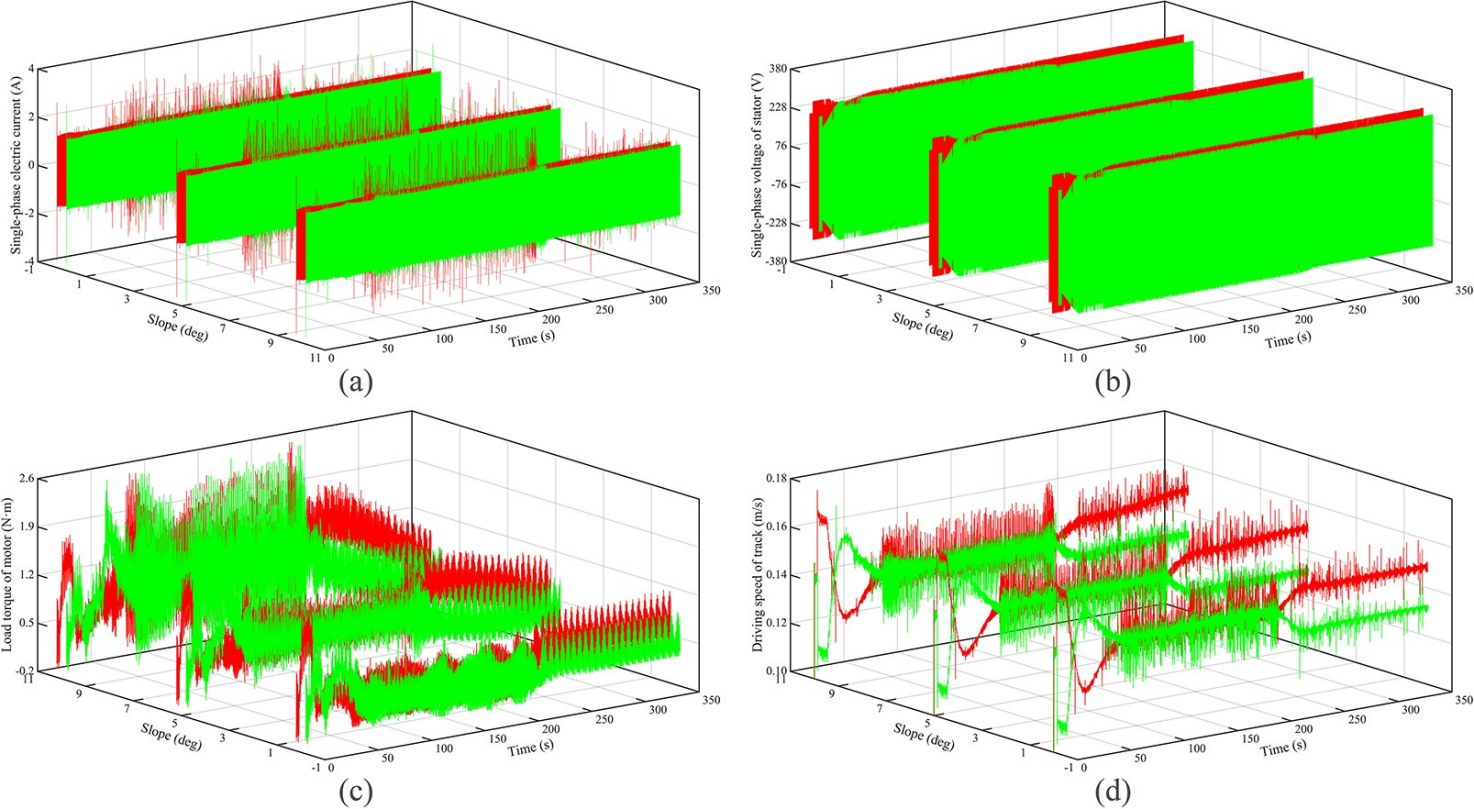
Electromechanical characteristics under climbing of soft ground.

The electromechanical characteristics of the autonomous walking system based on the fuzzy PID are shown in Fig 5. Red curves represent the left track, and green curves represent the right track. It can be seen that the motor current, voltage, and load torque all increase to varying degrees with an increase in the slope, but the theoretical driving speed of the crawler is reduced. Further, the motor current, voltage, and load torque are all greater than the values under hard ground conditions, the theoretical driving speed is lower than the value under hard ground conditions. The main reason is that with the increase of the slope, the load of the DTC along with the road surface increases, meanwhile, the pressure-sinkage effect of the crawler occurs, resulting in the motor load torque to increase significantly, and then the theoretical driving speed is further reduced. This causes the thermal power to increase, which in turn causes the current and voltage to increase to varying degrees. Secondly, it can be seen that the variation of the current, voltage, and load torque with the running time is consistent with that of hard ground. When the running time is greater than 200 s, DTC enters the curve tracking stage. As the slope increases, the current difference between the left and right crawler motors does not change significantly. However, relative to hard ground, the voltage difference and the load torque difference between the left and right tracks increase significantly. The main reason is that the sinking phenomenon becomes serious, leading to a significant increase in the rolling resistance of the crawler.

### Analysis under different turning radii

#### Hard ground conditions

**Fig 6:**
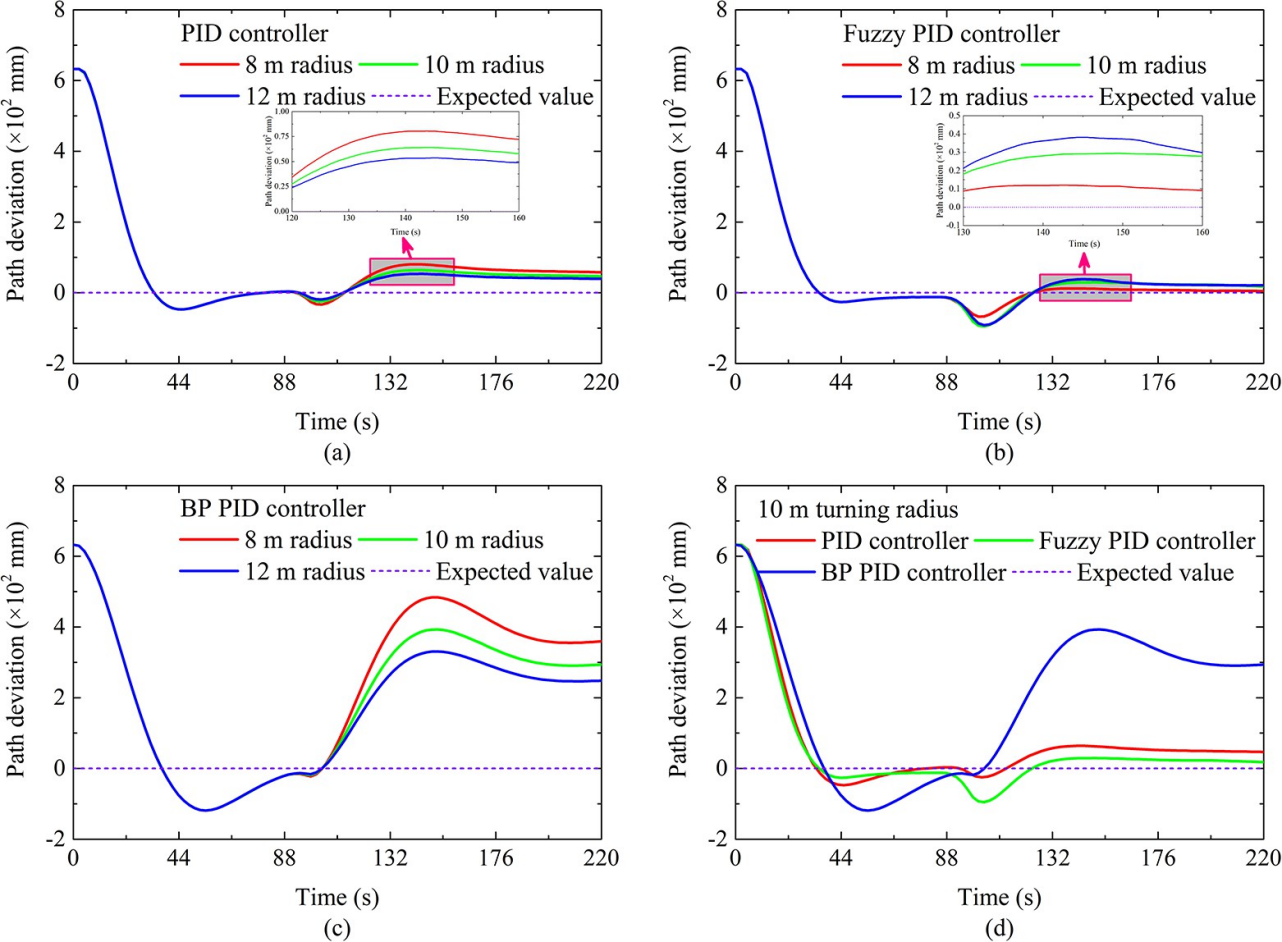
Autonomous walking performance under turning of hard ground.

The autonomous walking performance under hard ground conditions is shown in Fig 6. When the running time is from 0 s to 88 s, the DTC is in the AB straight-line autonomous walking stage. When the running time is greater than 88 s, the DTC enters the BC curve autonomous walking stage. As far as the overshoot of the BC section is concerned, it can be seen that the overshoot based on fuzzy PID is least affected by the turning radius. On the contrary, the overshoot based on BP PID is most affected by the turning radius, which is consistent with the results in Analysis under different slopes Section. As for the path deviation of the BC section, as the turning radius increases, the path deviation gradually decreases. The reason is that as the turn radius increases, the curvature of the preset trajectory becomes smaller, which causes the required steering adjustment intensity of DTC to decrease. In this way, under the same controller, the greater the turning radius, the better the reflected autonomous walking performance. Furthermore, it can be seen that the curve path deviation based on BP PID changes significantly, which indicates that the BP PID controller has a weaker perception of path deviation, which may be caused by the underfitting and the slow convergence speed of the BP neural network. In terms of curve response time, the curve response time based on fuzzy PID and PID is relatively small, but the response time based on BP PID is relatively long, which may be caused by the slow convergence speed of the BP neural network. Finally, the curve steady-state deviations based on PID and fuzzy PID are the smallest, which shows that the autonomous walking performance of the autonomous walking system based on PID and fuzzy PID is the best under this condition.

**Fig 7:**
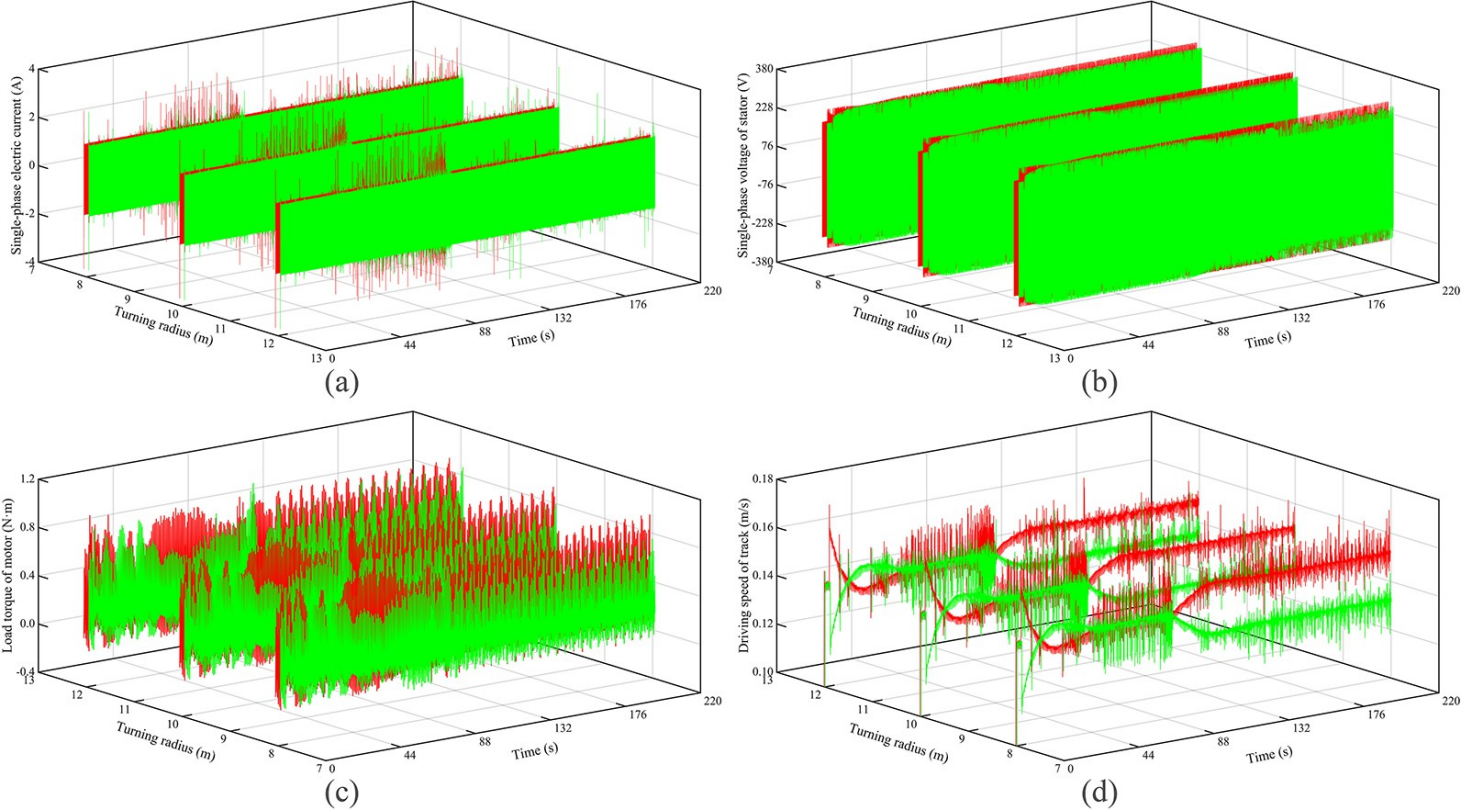
Electromechanical characteristics under turning of hard ground.

The electromechanical characteristics of the autonomous walking system based on the fuzzy PID are shown in Fig 7. Red curves represent the left track, and green curves represent the right track. It can be seen that as the turning radius increases, the motor current, voltage, load torque, and theoretical driving speed of the left crawler all decrease to varying degrees. The result of the right crawler is the opposite. The main reason is that with the increase of the turning radius, the power supply frequency of the left crawler motor is reduced, and the power supply frequency of the right crawler motor is increased, resulting in a decrease in the voltage and current of the left crawler, and therefore a reduction in the theoretical driving speed of the left crawler motor. At the same time, due to the reduction in the running speed of the left crawler, the dynamic load, the friction given by the ground, and the internal friction of the crawler are reduced, which in turn reduces the load torque, while the right crawler does the opposite.

### Soft ground conditions

**Fig 8:**
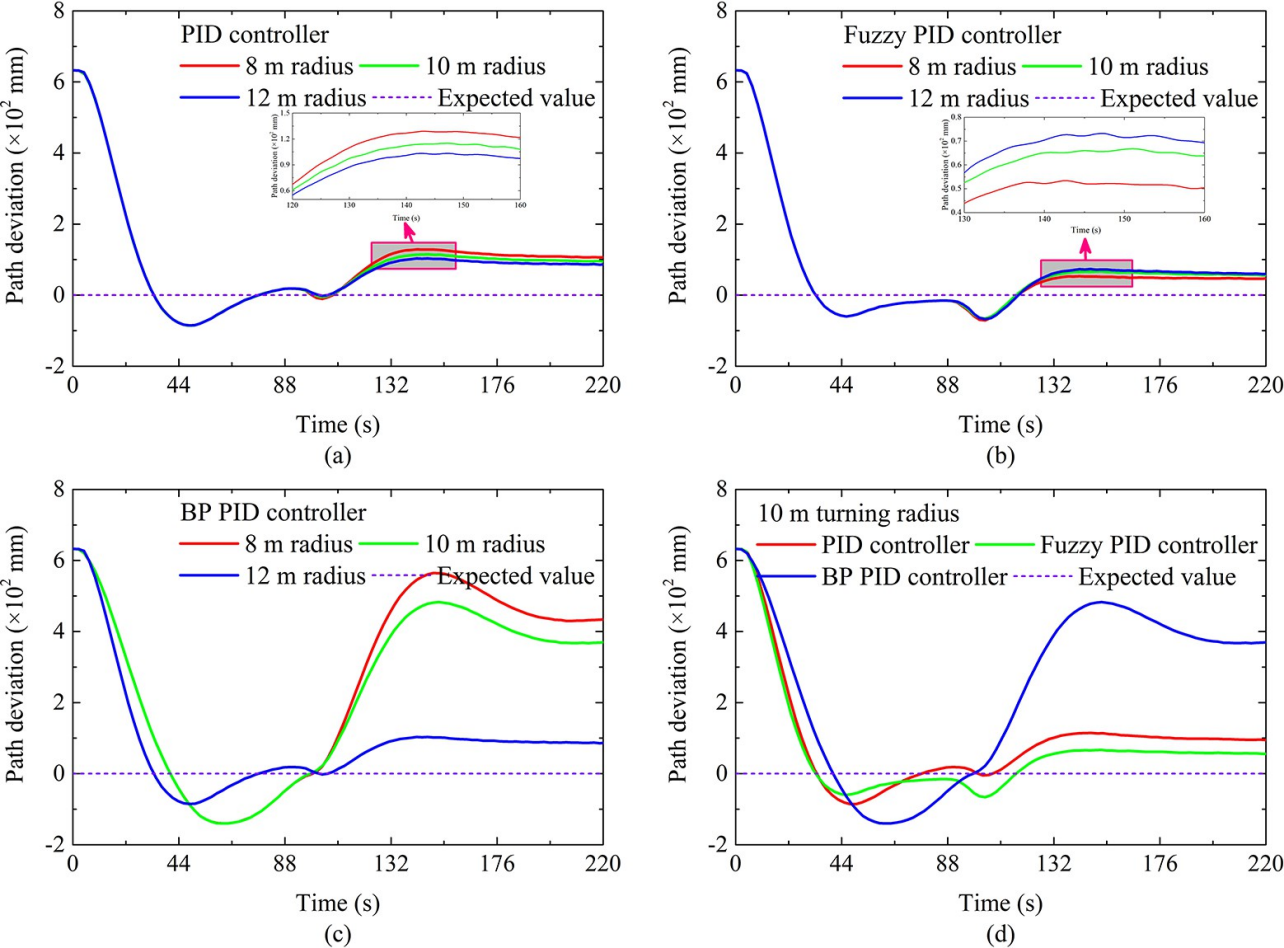
Autonomous walking performance under turning of soft ground.

The path tracking performance under soft ground conditions is shown in Fig 8. When the running time is from 0 s to 92 s, the DTC is in the AB straight-line autonomous walking stage. When the running time is greater than 92 s, the DTC enters the BC curve autonomous walking stage. As far as the overshoot of the BC section is concerned, it can be seen that the overshoot based on fuzzy PID is least affected by the turning radius. On the contrary, the overshoot based on BP PID is most affected by the turning radius, which is consistent with the results in Hard ground conditions of Analysis under different turning radii Section. The difference is that the overshoots are greater than the results of hard ground, the reason is that there is a lowering of the steering flexibility of the DTC due to the pressure-sinkage effect of the crawler under the soft ground condition, which makes DTC unable to adjust the driving status in time.

As for the path deviation of the BC section, as the turning radius increases, the path deviation gradually decreases. The reason is the same as that of the hard ground. Furthermore, it can be seen that the curve path deviation based on BP PID changes significantly, and when the turning radius is large, the path deviation changes more significantly, as shown in Fig 8(C), which shows that when the turning radius is large, the tracking performance of the autonomous walking system based on BP PID is significantly improved with a path deviation within 0.1 m. By comparing the response time, it can be seen that the autonomous walking performance at this time is better than the tracking performance of the other two autonomous walking systems. Therefore, when UME makes large-radius turns on soft roads, the autonomous walking system based on the BP PID can be given priority. In terms of curve response time, the curve response time based on fuzzy PID and PID is relatively small when the turning radius is small, which may be caused by the slow convergence speed of the BP neural network.

**Fig 9:**
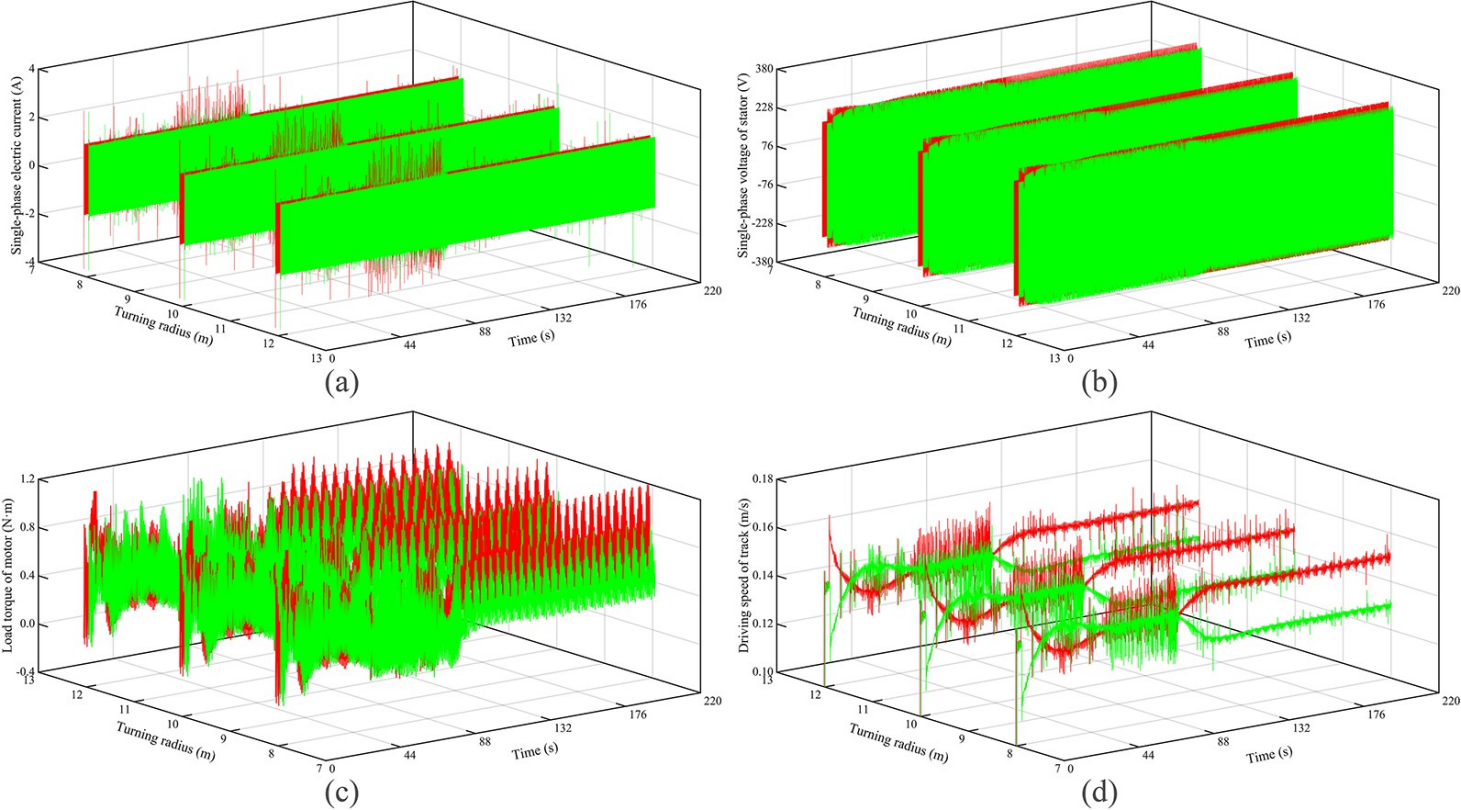
Electromechanical characteristics under turning of soft ground.

The electromechanical characteristics of the autonomous walking system based on the fuzzy PID are shown in Fig 9. Red curves represent the left track, and green curves represent the right track. It can be seen that as the turning radius increases, the variation of the motor current, voltage, load torque, and theoretical driving speed is consistent with the results in hard ground conditions. Further, the motor current, voltage, and load torque of the left crawler become larger compared to hard roads, but the theoretical driving speed is reduced. This is mainly because there is the pressure-sinkage effect of the crawler under the soft ground condition, which leads to a significant increase in the rolling resistance and the lateral resistance of the crawler. Therefore, the load torque of the motor is increased, and the theoretical driving speed is reduced. In the case of the same turning radius, the power supply frequency of the left crawler motors is increased, which in turn increases the motor current and voltage to varying degrees.

Besides, relative to hard ground, the voltage difference and the load torque difference between the left and right tracks are increased. The main reason is that the sinking phenomenon causes a significant increase in the load torque of the left crawler and a small change in the load torque of the right crawler. Therefore, the power supply frequency of the left crawler motor increases significantly, and the power supply frequency of the right crawler motor decreases significantly. Conversely, due to the significant increase in the load torque of the left crawler, the theoretical driving speed difference of the crawlers on both sides is reduced.

### Analysis of experiment

**Fig 10:**
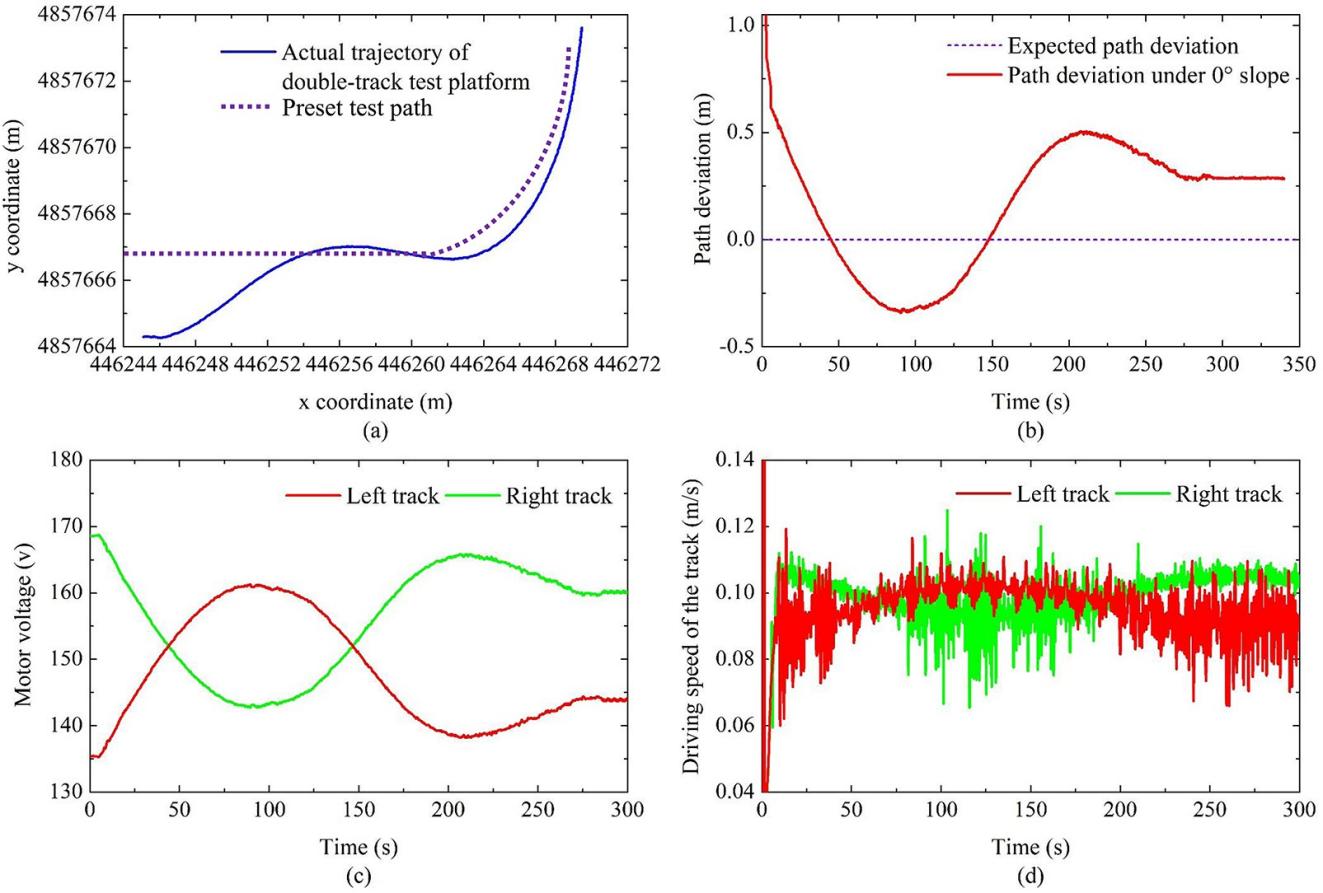
Results of experimental verification.

The autonomous walking test result based on fuzzy PID is shown in Fig 10. It can be seen that the double-track test platform can finally achieve curve tracking, which verifies the feasibility of the autonomous walking system, and the steady-state path deviation is 0.25 m, this one is greater than the corresponding simulation value. The reason may be that the long response time of the motor and the frequency inverter leads to the large inertia of the double-track test platform, and secondly, due to interference in the test environment, the positioning accuracy of the RTK positioning system decreases, which also makes the path deviation larger. During the autonomous walking process, the variation of the motor voltage and track running speed is consistent with the simulation result, that is, when the double-track test platform turns to travel, the voltage and running speed of the outer track increase to varying degrees, while the inner track is the opposite.

## Conclusions

1. Under hard ground conditions, the increase in slope causes the slippage between the crawler and the ground to become serious. As the turning radius increases, the power supply frequency of the left crawler motor is reduced, and therefore a reduction in the theoretical driving speed of the left crawler motor. Meanwhile, the dynamic load and friction is reduced, resulting in a reduction in the load torque.
2. Under soft ground conditions, the pressure-sinkage effect of the crawler occurs, and then the theoretical driving speed is lower than the value on hard ground. When UME makes large-radius turns on soft roads, the autonomous walking system based on the BP PID can be given priority with a path deviation within 0.1 m.
3. The curve response time based on BP PID is long, which may be caused by the slow convergence speed of the BP neural network.
4. The feasibility of the autonomous walking system is verified by the double-track test platform, and 0.25 m steady-state path deviation is greater than the corresponding simulation value. The reason may be that the long response time of the motor and the frequency inverter and interference in the test environment.

## Supporting information

S1 File Main parameters of mining double-track chassis and ground, fuzzy rule for fuzzy PID controller. (DOCX)

S2 File Membership function and double-track test platform. (DOCX)

## References

[1] Ranjan P, Wrat G, Bhola M, et al. A novel approach for the energy recovery and position control of a hybrid hydraulic excavator. ISA T 2020; 99: 387–402. doi: 10.1016/j.isatra.2019.08.066 31500901

[2] Sotiropoulos FE and Asada HH. Autonomous excavation of rocks using a gaussian process model and unscented Kalman filter. IEEE Robot Autom Let 2020; 5(2): 2491–2497.

[3] He Z, Nie L, Yin Z, et al. A two-layer controller for lateral path tracking control of autonomous vehicles. Sensors 2020; 20(13): 3689. doi: 10.3390/s20133689 32630197 PMC7374400

[4] Tian Y, Huang K, Cao X, et al. A hierarchical adaptive control framework of path tracking and roll stability for intelligent heavy vehicle with MPC. P I Mech Eng D–J Aut 2020; 234(13): 2933–2946.

[5] Qu X, Liang X, Hou Y, et al. Path-following control of unmanned surface vehicles with unknown dynamics and unmeasured velocities. J Mar Sci Technol 2021; 26(2): 395–407.

[6] Yu C, Liu C, Xiang X, et al. Line-of-sight guided time delay control for three-dimensional coupled path following of underactuated underwater vehicles with roll dynamics. Ocean Eng 2020; 207: 107410.

[7] Precup RE, Voisan EI, Petriu EM, et al. Grey wolf optimizer-based approaches to path planning and fuzzy logic-based tracking control for mobile robots. Int J Comput Commun 2020; 15(3): 3844.

[8] Qi ZF, Wang BZ, Qin YF, et al. Planar path following control for wave glider and experimental study. J Coastal Res 2020; 99: 16–20.

[9] Dogan MU, Guvenc U and Elmas C. Genetic PI based model and path tracking control of four traction electrical vehicle. Electr Eng 2020; 102(4): 2059–2073.

[10] Shi Y, Elara MR, Le AV, et al. Path tracking control of self-reconfigurable robot hTetro with four differential drive units. IEEE Robot Autom Let 2020; 5(3): 3998–4005.

[11] Salt J, Alcaina J, Cuenca Á, et al. Multirate control strategies for avoiding sample losses. Application to UGV path tracking. ISA T 2020; 101: 130–146. 10.1016/j.isatra.2020.01.025 31964541

[12] Rayguru MM, Elara MR, Balakrishnan R, et al. A path tracking strategy for car like robots with sensor unpredictability and measurement errors. Sensors 2020; 20(11): 3077. doi: 10.3390/s20113077 32485928 PMC7308858

[13] Rao Y and Yang F. Research on path tracking algorithm of autopilot vehicle based on image processing. Int J Pattern Recogn 2019; 34(5): 2054013.

[14] Kim E, Fan S, Bose N, et al. Current estimation and path following for an autonomous underwater vehicle (AUV) by using a high-gain observer based on an AUV dynamic model. Int J Control Autom 2019; 52(21): 218–223.

[15] Kumar V, Nakra BC and Mittal AP. A review of classical and fuzzy PID controllers. Int J Intell Control Syst 2011; 16(3): 170–181.

[16] Sun L, You F. Machine learning and data-driven techniques for the control of smart power generation systems: An uncertainty handling perspective. Engineering 2021; 7(9): 1239–1247.

[17] Yan C, Li M, Liu W, et al. Improved adaptive genetic algorithm for the vehicle Insurance Fraud Identification Model based on a BP Neural Network. Theor Compu Sci 2020; 817: 12–23.

[18] Wu G, Wang G, Bi Q, et al. Research on unmanned electric shovel autonomous driving path tracking control based on improved pure tracking and fuzzy control. J Field Robot 2023; 40(7): 1739–1753.

[19] Fang Y, Wang S, Bi Q, et al. Research on path planning and trajectory tracking of an unmanned electric shovel based on improved APF and preview deviation fuzzy control. Machines 2022; 10(8): 707.

[20] Guan W, Wang S, Chen Z, et al. An automatic alignment method for discharge arm of mobile crushing station based on binocular vision and fuzzy control. T I Meas Control 2022; 45(6): 1001–1020.

[21] Chen Z, Xue D, Guan W, et al. Research on the disturbance behaviour of the track chassis to the sand-gravel pavement during the steering process of the electric shovel based on DEM. Adv Powder Technol 2022; 33(9): 103731.

[22] Chen Z, Xue D, Wang G, et al. Simulation and optimization of the tracked chassis performance of electric shovel based on DEM-MBD. Powder Technol 2021; 390: 428–441.

[23] Zhang G, Wang Y, Fan Y, et al. Adaptive inverse control based on Kriging algorithm and Lyapunov theory of crawler electromechanical system. Complexity 2018; 2018: 1872943.

[24] Wang S, Zhang SB, Ma RD, et al. Remote control system based on the Internet and machine vision for tracked vehicles. J Mech Sci Technol 2018; 32(3): 1317–1331.

[25] Wan P, Zou H, Wang K, et al. Research on hot deformation behavior of Zr-4 alloy based on PSO-BP artificial neural network. J Alloy Compd 2020; 826: 154047.

[26] Bekker M. Theory of land locomotion–The mechanics of vehicle mobility. Ann Arbor, Michigan: The University of Michigan Press, 1956.

