# Supplementary material for: Research on autonomous walking performance and electromechanical characteristics of mining double-track chassis
Source: PLoS One. 2024 Dec 3;19(12):e0312096. doi: 10.1371/journal.pone.0312096 (PMC11614219; doi:10.1371/journal.pone.0312096)
Supplement: S1 File — (DOCX) [file pone.0312096.s001.docx]

**Table S1. Main parameters of mining double-track chassis and ground.**

| Parameters | Value | Parameters | Value |
| --- | --- | --- | --- |
| DTC weight *m* (kg) | 450 | Stiffness *k* (N/mm) | 10000 |
| Track centroid distance *B* (m) | 0.93 | Damping coefficient *c* (N·s/mm) | 10 |
| Winding resistances of the stator *R_s_* (Ω) | 35 | Dynamic friction coefficient *μ_d_* | 0.7 |
| Gear transmission ratio *i* | 100 | Cohesive deformation modulus *k_c_* (N·mm^-n-1^) | 4.76×10^-4^ |
| Track ground length *l* (m) | 0.881 | Friction deformation modulus *k_φ_* (N·mm^-n-2^) | 7.66×10^-4^ |
| Moment of inertia of DTC *J_O_* (kg·m²) | 115.03 | Soil deformation index *n* | 0.2 |
| Density (kg/m^3^) | 7850 | Cohesion *C* (Pa) | 1.04×10^-3^ |
| Young’s modulus (Pa) | 2×10^11^ | Angle of internal shearing resistance *φ* (deg) | 28 |
| Poisson’s ratio | 0.285 | Shear deformation modulus *K* | 25 |

**Table S2. Fuzzy rule for fuzzy PID controller.**

| Inputs | | *e*(*t*) |  |  |  |  |  |  |
| --- | --- | --- | --- | --- | --- | --- | --- | --- |
|  |  | NB | NM | NS | ZO | PS | PM | PB |
| *ec*(*t*) | NB | *K_P_*_1_*K_i_*_1_*K_d_*_2_ | *K_P_*_2_*K_i_*_2_*K_d_*_1_ | *K_P_*_1_*K_i_*_5_*K_d_*_5_ | *K_P_*_2_*K_i_*_2_*K_d_*_3_ | *K_P_*_1_*K_i_*_1_*K_d_*_1_ | *K_P_*_1_*K_i_*_4_*K_d_*_5_ | *K_P_*_1_*K_i_*_4_*K_d_*_4_ |
|  | NM | *K_P_*_3_*K_i_*_1_*K_d_*_2_ | *K_P_*_3_*K_i_*_2_*K_d_*_3_ | *K_P_*_2_*K_i_*_5_*K_d_*_5_ | *K_P_*_3_*K_i_*_2_*K_d_*_3_ | *K_P_*_3_*K_i_*_1_*K_d_*_3_ | *K_P_*_1_*K_i_*_4_*K_d_*_4_ | *K_P_*_2_*K_i_*_4_*K_d_*_4_ |
|  | NS | *K_P_*_5_*K_i_*_1_*K_d_*_3_ | *K_P_*_5_*K_i_*_2_*K_d_*_3_ | *K_P_*_1_*K_i_*_5_*K_d_*_1_ | *K_P_*_3_*K_i_*_2_*K_d_*_3_ | *K_P_*_5_*K_i_*_1_*K_d_*_4_ | *K_P_*_5_*K_i_*_4_*K_d_*_3_ | *K_P_*_4_*K_i_*_5_*K_d_*_2_ |
|  | ZO | *K_P_*_4_*K_i_*_1_*K_d_*_3_ | *K_P_*_5_*K_i_*_2_*K_d_*_4_ | *K_P_*_3_*K_i_*_5_*K_d_*_3_ | *K_P_*_4_*K_i_*_2_*K_d_*_5_ | *K_P_*_4_*K_i_*_1_*K_d_*_4_ | *K_P_*_3_*K_i_*_4_*K_d_*_5_ | *K_P_*_3_*K_i_*_5_*K_d_*_2_ |
|  | PS | *K_P_*_5_*K_i_*_1_*K_d_*_5_ | *K_P_*_4_*K_i_*_2_*K_d_*_4_ | *K_P_*_3_*K_i_*_5_*K_d_*_5_ | *K_P_*_4_*K_i_*_2_*K_d_*_5_ | *K_P_*_5_*K_i_*_1_*K_d_*_5_ | *K_P_*_5_*K_i_*_4_*K_d_*_4_ | *K_P_*_2_*K_i_*_4_*K_d_*_5_ |
|  | PM | *K_P_*_4_*K_i_*_1_*K_d_*_2_ | *K_P_*_5_*K_i_*_2_*K_d_*_3_ | *K_P_*_1_*K_i_*_5_*K_d_*_2_ | *K_P_*_3_*K_i_*_2_*K_d_*_4_ | *K_P_*_5_*K_i_*_1_*K_d_*_4_ | *K_P_*_3_*K_i_*_5_*K_d_*_3_ | *K_P_*_3_*K_i_*_5_*K_d_*_4_ |
|  | PB | *K_P_*_5_*K_i_*_1_*K_d_*_4_ | *K_P_*_5_*K_i_*_2_*K_d_*_5_ | *K_P_*_1_*K_i_*_5_*K_d_*_4_ | *K_P_*_3_*K_i_*_2_*K_d_*_3_ | *K_P_*_5_*K_i_*_1_*K_d_*_4_ | *K_P_*_3_*K_i_*_5_*K_d_*_3_ | *K_P_*_5_*K_i_*_5_*K_d_*_4_ |
