# Supplementary material for: Research on autonomous walking performance and electromechanical characteristics of mining double-track chassis
Source: PLoS One. 2024 Dec 3;19(12):e0312096. doi: 10.1371/journal.pone.0312096 (PMC11614219; doi:10.1371/journal.pone.0312096)
Supplement: S2 File — (DOCX) [file pone.0312096.s002.docx]

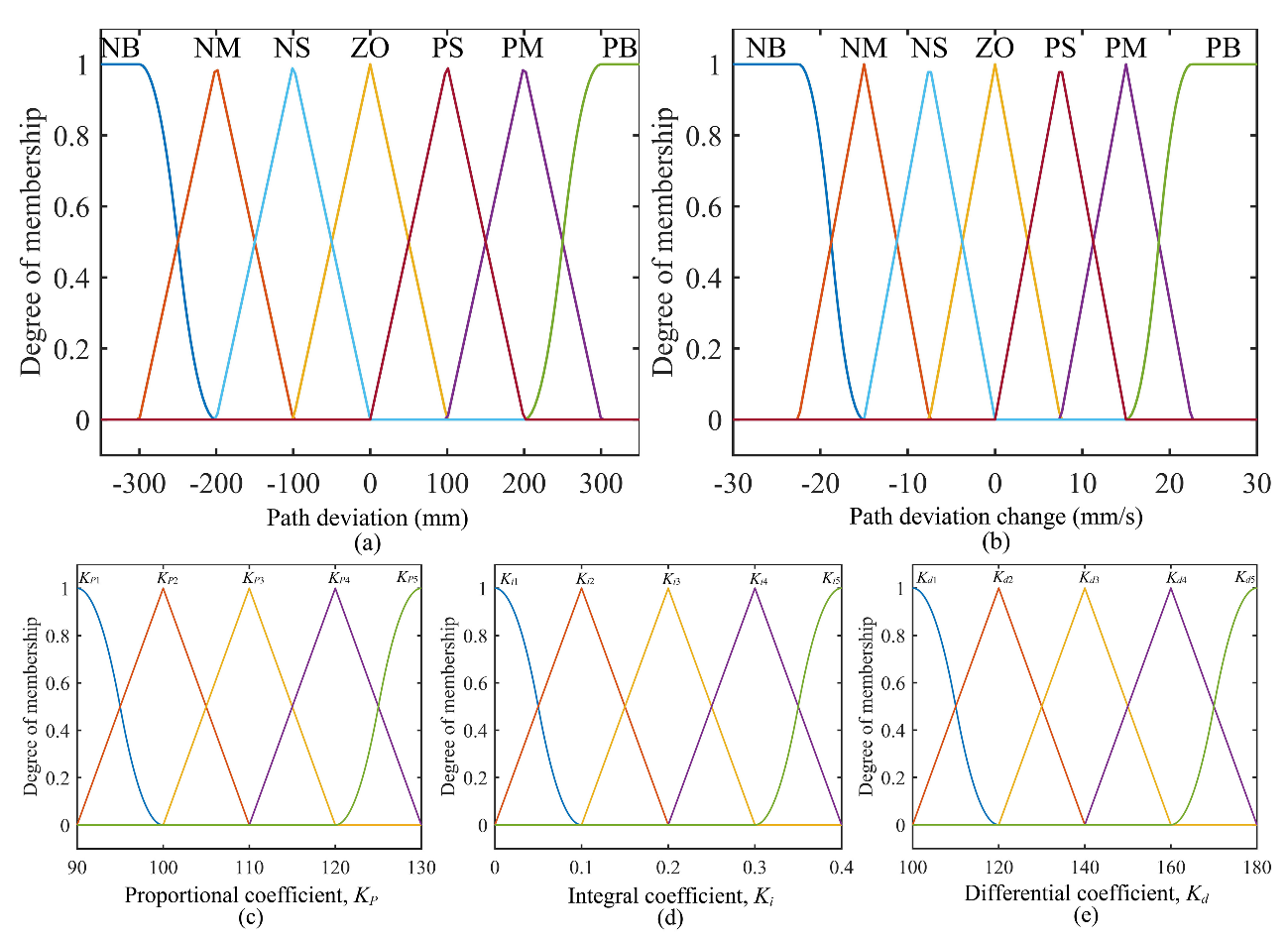


**Fig S1.** **Membership function.** (a) Path deviation. (b) Path deviation change. (c) Proportional coefficient. (d) Integral coefficient. (e) Differential coefficient.


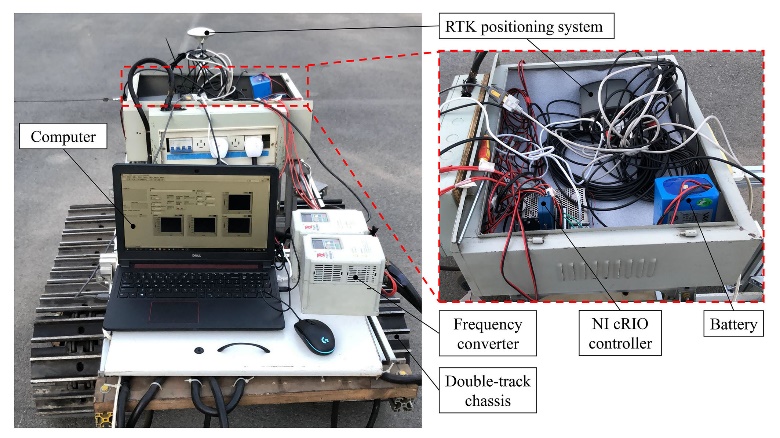


**Fig S2. Schematic diagram of double-track test platform.**
